# *Interleukin 10* gene rs1800896 polymorphism is associated with the risk of prostate cancer

**DOI:** 10.18632/oncotarget.19857

**Published:** 2017-08-03

**Authors:** Hao Chen, Jilei Tang, Nan Shen, Kewei Ren

**Affiliations:** ^1^ Department of Urology, The First Hospital of Jiaxing, Jiaxing 314001, China; ^2^ Department of Orthopedics, Qidong People’s Hospital, Nantong 226200, China; ^3^ Department of Clinical Pharmacy, The Affiliated Jiangyin Hospital of Southeast University Medical School, Jiangyin 214400, China; ^4^ Department of Orthopedics, The Affiliated Jiangyin Hospital of Southeast University Medical School, Jiangyin 214400, China

**Keywords:** interleukin-10, polymorphism, prostate cancer, meta-analysis

## Abstract

Numerous studies have uncovered the association of Interleukin-10 (*IL-10*) gene rs1800896 polymorphism with the risk of prostate cancer (PCa); however, their conclusions were inconsistent. Therefore, we conducted this meta-analysis to evaluate the role of *IL-10* rs1800896 polymorphism in the risk of PCa. 16 eligible studies in 15 articles involving 6,301 cases and 6,510 controls were identified by researching PubMed, Google, CNKI, and EMBASE up to April 1, 2017. Our results revealed that *IL-10* rs1800896 polymorphism was associated with the decreased risk of PCa under the homozygous model. Subgroup analysis by ethnicity revealed that rs1800896 polymorphism decreased the risk of PCa among Caucasians. In conclusion, *IL-10* gene rs1800896 polymorphism is associated with the decreased risk of PCa. Larger studies with more diverse ethnic populations are needed to confirm these results.

## INTRODUCTION

Prostate cancer (PCa) is the most common malignancy diagnosed in men and the second leading cause of cancer-related death for men in the United States [1]. The American Cancer Society estimates that 161,360 new PCa cases and 26,730 PCa deaths will occur in 2017 [2]. Widespread use of prostate-specific antigen (PSA) screening could reduce mortality of PCa. However, PSA screening may contribute to overdiagnosis and overtreatment [3]. New predictive biomarkers are becoming an urgent need, such as single nucleotide polymorphism (SNP) [4]. Remarkably, Xu et al. found that SNPs of four genes in the inflammation pathway, including interleukin-10 (*IL-10*) could significantly predict the risk of PCa [5].

*IL-10* is a pleiotropic cytokine that modulates the function of several adaptive immunity-related cells [6]. *IL-10* is known to suppress the functions of both T lymphocytes and macrophages, working as a general dampener of the immune and inflammatory responses [7]. Huang et al. found that *IL-10* could suppress metastasis and growth of the tumor by inhibiting macrophage-derived angiogenic factors *in vivo* [8]. Richter et al. also found that *IL-10* transfected into Chinese hamster ovary cells prevents tumor growth and macrophage infiltration [9]. In addition, overexpression of *IL-10* was observed in PCa tissue by the analysis of type T1 and T2 cytokines in patients with PCa [10]. Yu et al. also found *IL-10* decreased stemness of human prostate cancer cells *in vitro* [11]. Based on these observations, *IL-10* may provide insights about screening and therapeutic intervention on neoplastic patients.

Kingo et al. identified that *IL-10* rs1800896 polymorphism may have an influence on *IL-10* mRNA expression and the expression of *IL-10 in vitro* [12]. Recently, a host of studies [5, 13–26] attached importance to evaluating whether *IL-10* rs1800896 polymorphism had effects on the risk of PCa. Some studies yielded relations between *IL-10* rs1800896 polymorphism and PCa risk [16, 19, 22], but other studies could not [5, 13-15, 17, 18, 20, 21, 23-26]. Small sample sizes, low statistical power, and clinical heterogeneity may contribute to these conflicting results. Therefore, we conducted this meta-analysis to review available evidence and evaluate the effects of rs1800896 polymorphism in *IL-10* gene on PCa susceptibility in the overall population.

## RESULTS

### Characteristics of the included studies

**Figure 1:**
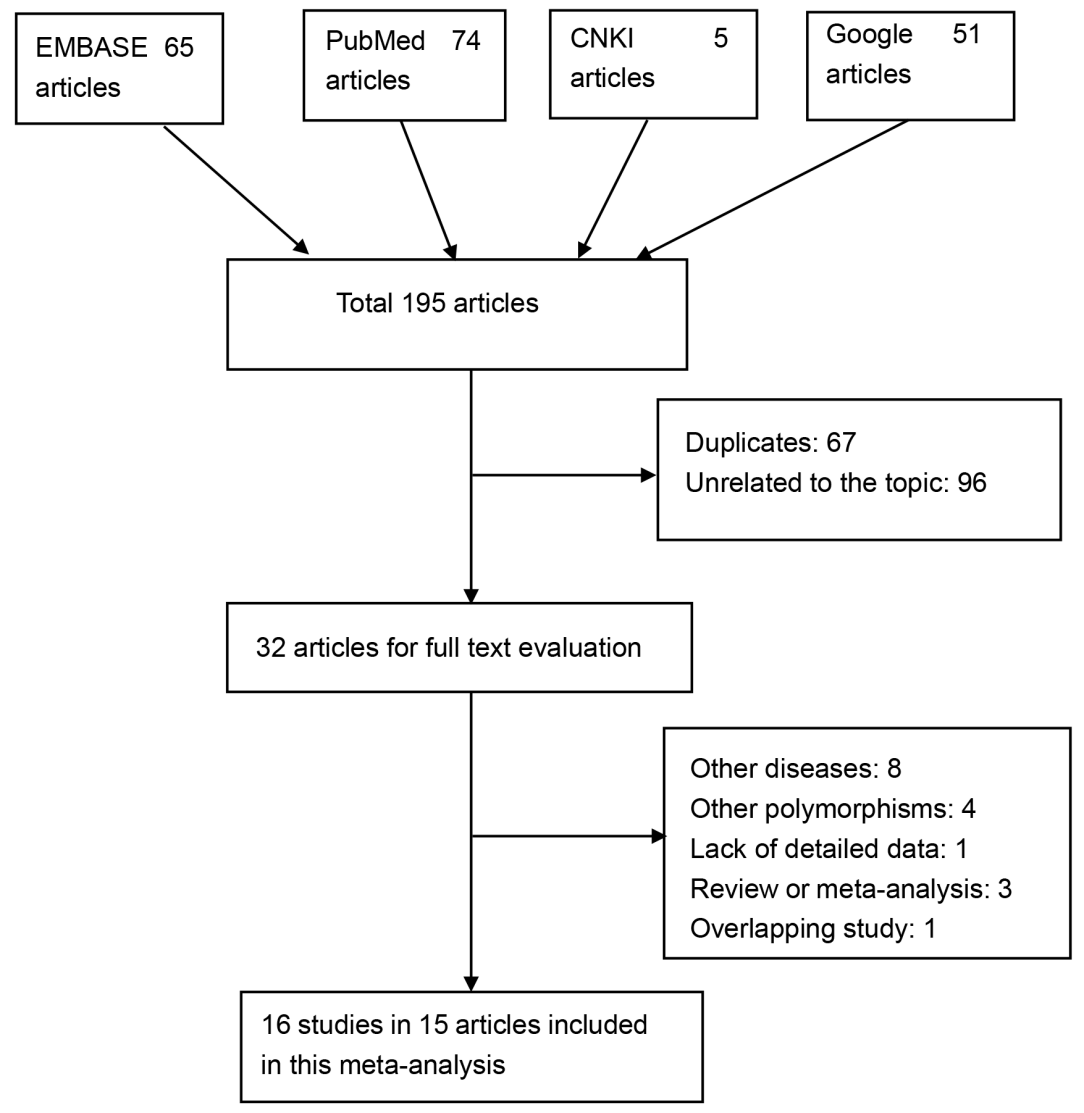
Selection of eligible publications included in this meta-analysis

We used the following searched terms like prostate cancer, prostatic neoplasm, prostate carcinoma, prostate neoplasms, polymorphism, polymorphisms, SNP, SNPs, rs1800896, Interleukin 10, *IL-10* and IL10 to identify relevant articles. Totally 195 articles were identified after an electronic and manual search (PubMed, 74 articles; EMBASE, 65 articles; CNKI, 5 articles; Google, 51 articles). Then 67 duplicates and 96 articles unrelated to this topic were removed. 32 articles remained for further full-text review. 17 articles were excluded due to the following reasons: eight articles investigated other diseases rather than PCa; three articles investigated other polymorphisms of *IL-10* gene; one article did not provide detailed genotyping data; three articles were reviews or meta-analyses; one article was excluded due to overlapping information with another study. One article was divided into two studies because it contains two different racial groups. Finally, 16 eligible studies in 15 articles with 6,301 patients and 6,510 controls were included in this meta-analysis. The selection for eligible studies was presented in Figure 1. Most of the included studies [5, 15, 16, 18-22, 24, 25] were population-based studies, focusing on Caucasians. In addition, we found that five studies [13-15, 21, 27] did not conform to Hardly-Weinberg equilibrium (HWE). The phenomenon may be partly attributed to genotyping error, population stratification and selection bias in the recruitment of controls. Moreover, the Newcastle-Ottawa Scale (NOS) scores of all included studies ranged from 5 to 7 stars, suggesting that these studies were of high methodological quality. The detailed characteristics of included studies are listed in Table 1.

**Table 1:** Characteristics of included studies for the association between *IL-10* rs1800896 polymorphism and prostate cancer

Author+year	Country	Ethnicity	SOC	Genotyping method	Case			Control			HWE	NOS
					GG	GA	AA	GG	GA	AA		
McCarron2002	UK	Caucasian	PB	PCR	56	113	78	57	120	46	Yes	7
Xu2005	Sweden	Caucasian	PB	MassARRAY	306	689	388	187	390	203	Yes	6
Michaud2006	US	Mixed	PB	Taqman	290	599	356	383	857	523	Yes	7
Faupel-Badger2008	US	Caucasian	PB	Taqman	85	251	173	73	194	115	Yes	6
Omrani2008	Iran	Caucasian	HB	PCR	5	31	5	2	98	3	No	5
Zabaleta2008	US	Caucasian	HB	Taqman	126	239	110	86	206	102	Yes	6
Zabaleta2008	US	African	HB	Taqman	7	38	21	13	74	42	No	6
Kesarwani2009	India	Caucasian	PB	PCR	12	78	69	45	103	111	No	6
Wang2009	US	Caucasian	PB	Taqman	56	130	69	83	117	57	Yes	6
Liu2010	China	Asian	PB	PCR	4	36	222	3	27	240	Yes	7
VanCleave2010	US	African	HB	Taqman	75	95	22	288	280	92	Yes	7
Niu2011	China	Asian	HB	N/A	18	56	24	20	26	42	No	6
Dluzniewski2012	US	Caucasian	PB	MassARRAY	100	212	146	104	242	112	Yes	7
Lanni2013	Italy	Caucasian	PB	Taqman	18	74	79	28	43	25	Yes	6
Horvat2015	Croatia	Caucasian	HB	Taqman	24	59	37	24	54	42	Yes	6
Winchester2017	US	Caucasian	PB	MassARRAY	136	305	179	140	254	134	No	7

SOC, source of controls; PB, population-based controls; HB, hospital-based controls; HWE, hardy-weinberg equilibrium; NOS, newcastle-ottawa scale.

### Quantitative analysis

**Figure 2:**
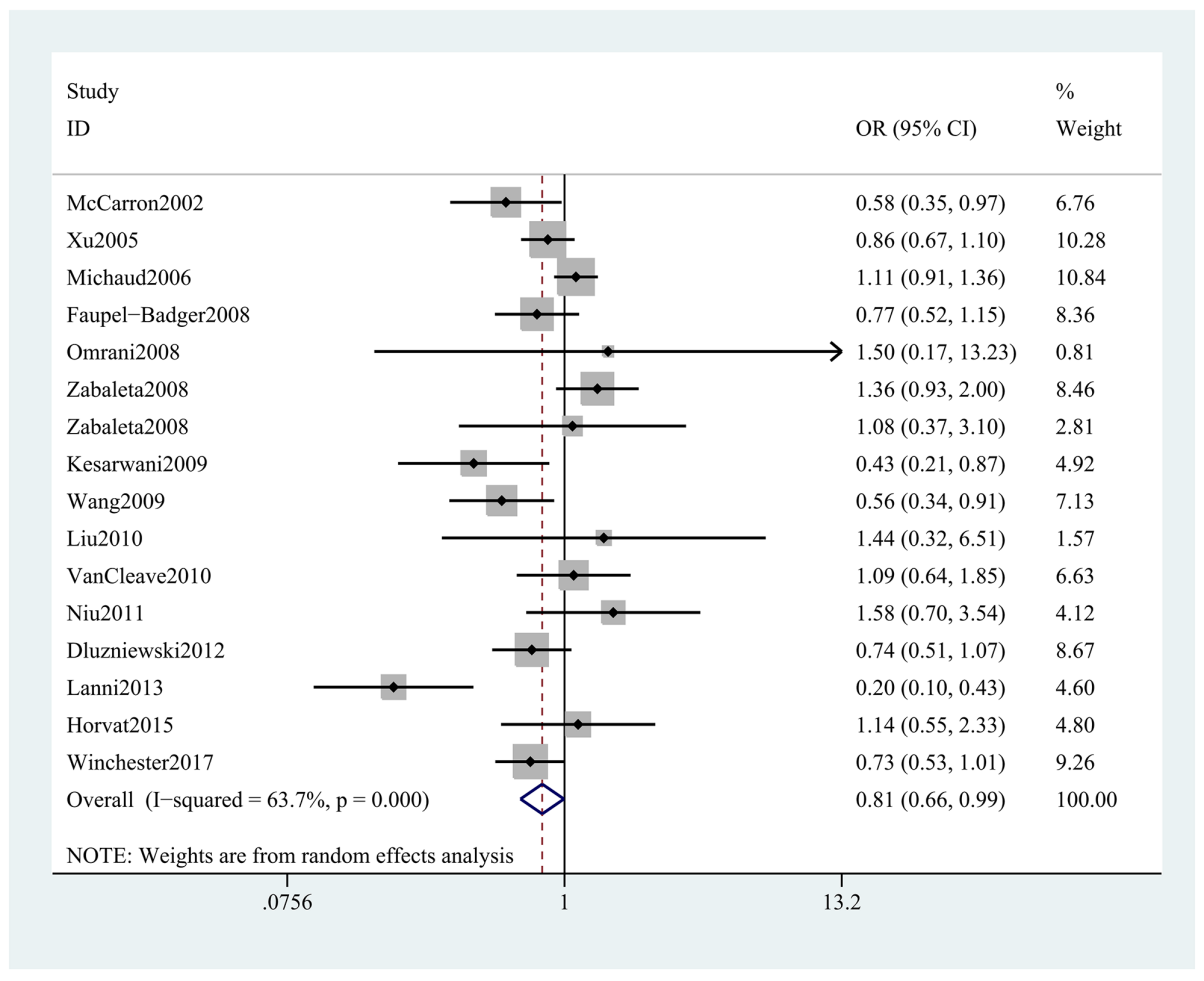
Forest plot shows odds ratio for the associations between *IL-10* rs1800896 polymorphism and PCa risk (GG vs. AA)

**Figure 3:**
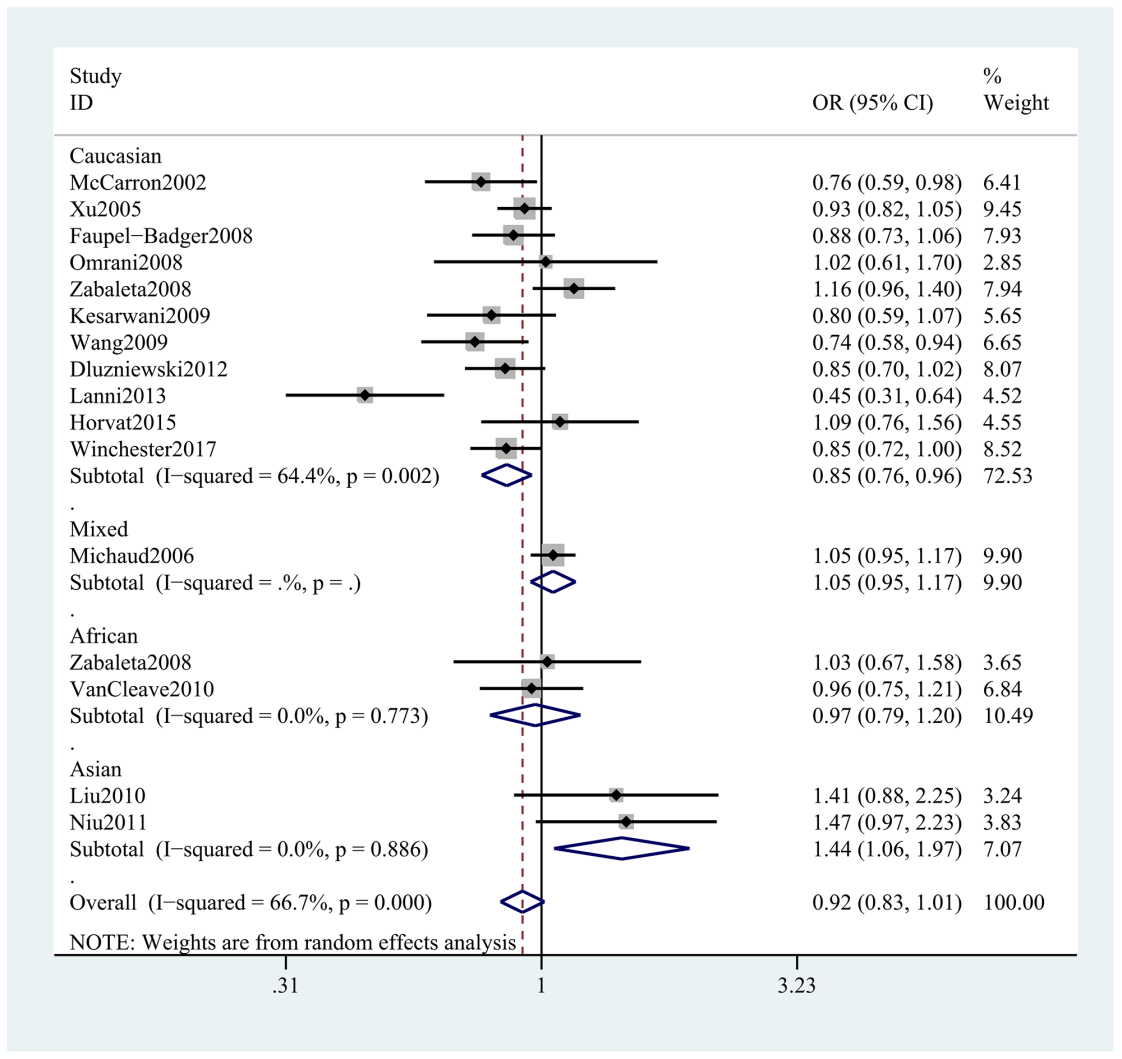
Stratification analyses of ethnicity between *IL-10* rs1800896 polymorphism and PCa risk (G vs. A)

The findings of our meta-analysis uncovering the association between *IL-10* rs1800896 polymorphism and PCa was summarized in Tables 2 and 3. The meta-analysis revealed that individuals carrying GG genotypes had a 19% lower risk of developing PCa compared to those who carried AA genotypes in the overall population (GG vs. AA: OR, 0.81; 95% CI, 0.66–0.99, *P* = 0.044, Figure 2). Stratification analyses were conducted according to ethnicity, HWE, SOC and genotyping method. Ethnicity subgroup analysis indicated that the risk allele of rs1800896 in the *IL-10* gene was associated with the decreased risk of PCa among Caucasians in most of the comparisons (G vs. A: OR, 0.85; 95% CI, 0.76–0.96, *P* = 0.007, Figure 3), while the opposite result was found among Asians in the allele model (G vs. A: OR, 1.44; 95% CI, 1.06–1.97, *P* = 0.020, Figure 3) and dominant model. In addition, we failed to find this significant association among Africans under five models (Table 3). Stratification analyses by SOC and genotyping method suggested that population-based studies or studies using MassARRAY method were easy to find the associations between *IL-10* rs1800896 polymorphism and risk of PCa. However, when stratified by HWE, there is no significant association in the HWE-positive groups or HWE-negative groups. Obviously, these studies deviating from HWE have effects on the overall results.

**Table 2:** Meta-analysis for the effect of rs1800896 on the risk of prostate cancer

Genetic model	Statistics		Heterogeneity		Publication bias	
	OR(95%CI)	*P*	*P*_heterogeneity_	I^2^ (%)	*P*_begg_	*P*_Egger_
Allele (G vs. A)	0.92(0.83,1.01)	0.089	<0.001	66.7	0.857	0.590
Dominant (GG+GA vs. AA)	0.92(0.79,1.08)	0.319	<0.001	67.2	0.787	0.742
Recessive (GG vs. GA+AA)	0.85(0.72,1.00)	0.052	<0.001	62.6	0.928	0.418
Homozygous (GG vs. AA)	**0.81(0.66,0.99)**	0.044	<0.001	63.7	0.719	0.399
Heterozygous (GA vs. AA)	1.02(0.85,1.23)	0.814	<0.001	72.0	0.787	0.938

*Bold values are statistically significant (*P* < 0.05).

**Table 3:** Summary of the subgroup analyses in this meta-analysis

Comparison	Category	Category	Studies	OR (95% CI)	*P*-value	*P* for heterogeneity
G vs. A	Ethnicity	Caucasian	11	**0.85(0.76,0.96)**	0.007	0.002
		Mixed	1	1.05(0.95,1.17)	0.312	N/A
		African	2	0.97(0.79,1.20)	0.786	0.773
		Asian	2	**1.44(1.06,1.97)**	0.020	0.886
	SOC	PB	10	**0.85(0.75,0.96)**	0.008	<0.001
		HB	6	1.10(0.97,1.24)	0.125	<0.001
	HWE	Positive	11	0.90(0.80,1.02)	0.097	<0.001
		Negative	5	0.96(0.79,1.16)	0.654	0.128
	Method	PCR	4	0.91(0.70,1.17)	0.452	0.117
		MassARRAY	3	**0.89(0.81,0.97)**	0.006	0.625
		Taqman	8	0.90(0.77,1.07)	0.230	<0.001
		N/A	1	1.47(0.97,2.23)	0.066	N/A
GG+GA vs. AA	Ethnicity	Caucasian	11	**0.81(0.69,0.95)**	0.009	0.014
		Mixed	1	1.05(0.90,1.24)	0.525	N/A
		African	2	1.16(0.79,1.72)	0.445	0.643
		Asian	2	**1.96(1.02,3.78)**	0.043	0.102
	SOC	PB	10	**0.83(0.71,0.97)**	0.021	0.004
		HB	6	1.23(0.86,1.78)	0.261	0.028
	HWE	Positive	11	0.89(0.75,1.05)	0.163	0.001
		Negative	5	1.04(0.64,1.68)	0.870	0.002
	Method	PCR	4	0.79(0.46,1.37)	0.404	0.009
		MassARRAY	3	**0.83(0.72,0.96)**	0.012	0.333
		Taqman	8	0.94(0.77,1.14)	0.527	0.027
		N/A	1	**2.82(1.51,5.24)**	0.001	N/A
GG vs.GA+ AA	Ethnicity	Caucasian	11	0.81(0.65,1.00)	0.054	<0.001
		Mixed	1	1.09(0.92,1.30)	0.309	N/A
		African	2	0.85(0.62,1.16)	0.303	0.638
		Asian	2	0.85(0.45,1.63)	0.628	0.488
	SOC	PB HB	10 6	**0.78(0.64,0.95)** 1.06(0.77,1.46)	0.012 0.735	0.001 <0.001
	HWE	Positive	11	0.87(0.73,1.04)	0.129	0.003
		Negative	5	0.82(0.49,1.39)	0.471	0.026
	Method	PCR	4	1.01(0.43,2.39)	0.982	0.010
		MassARRAY	3	0.87(0.76,1.01)	0.070	0.589
		Taqman	8	0.84(0.65,1.09)	0.189	0.001
		N/A	1	0.76(0.37,1.56)	0.462	N/A
GG vs. AA	Ethnicity	Caucasian	11	**0.71(0.56,0.90)**	0.005	0.001
		Mixed	1	1.11(0.91,1.36)	0.305	N/A
		African	2	1.09(0.68,1.75)	0.731	0.985
		Asian	2	1.58(0.70,3.54)	0.233	0.919
	SOC	PB	10	**0.69(0.54,0.88)**	0.002	<0.001
		HB	6	1.26(0.97,1.64)	0.078	0.969
	HWE	Positive	11	0.81(0.64,1.03)	0.085	<0.001
		Negative	5	0.81(0.52,1.25)	0.336	0.164
	Method	PCR	4	**0.58(0.39,0.86)**	0.007	0.417
		MassARRAY	3	**0.79(0.66,0.94)**	0.008	0.676
		Taqman	8	0.84(0.60,1.17)	0.296	<0.001
		N/A	1	1.58(0.70,3.54)	0.272	N/A
GA vs. AA	Ethnicity	Caucasian	11	**0.90(0.72,1.11)**	0.328	<0.001
		Mixed	1	1.03(0.87,1.22)	0.761	N/A
		African	2	1.25(0.83,1.88)	0.280	0.448
		Asian	2	2.27(0.89,5.82)	0.087	0.030
	SOC	PB	10	**0.94(0.77,1.14)**	0.508	<0.001
		HB	6	1.26(0.79,1.99)	0.329	0.004
	HWE	Positive	11	0.92(0.79,1.07)	0.265	0.021
		Negative	5	1.35(0.80,2.28)	0.257	0.002
	Method	PCR	4	0.81(0.44,1.51)	0.513	0.003
		Taqman	3	1.00(0.64,1.58)	0.989	<0.001
		MassARRAY	8	0.99(0.87,1.13)	0.907	0.350
		N/A	1	**3.77(1.90,7.47)**	<0.001	N/A

SOC, source of controls; PB, population-based controls; HB, hospital-based controls; HWE: hardy-weinberg equilibrium.

### Heterogeneity and publication bias

**Figure 4:**
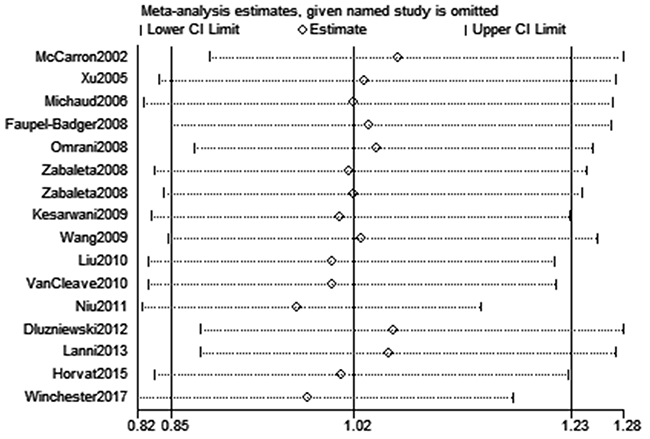
Sensitivity analysis for the association between *IL-10* gene rs1800896 polymorphism and PCa risk (GA vs. AA)

**Figure 5:**
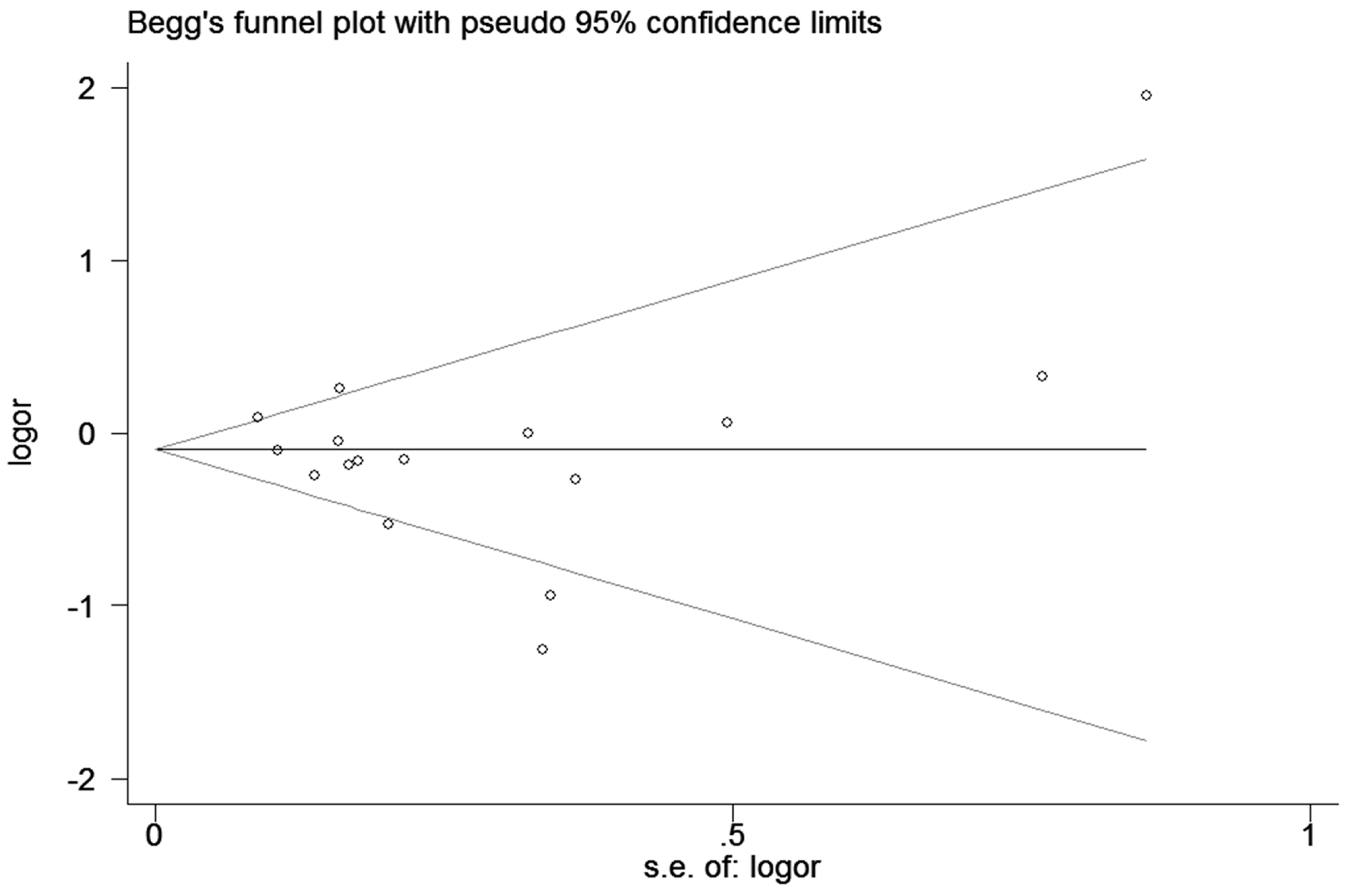
Begg’s tests between *IL-10* rs1800896 polymorphism and PCa risk (GG vs. GA+AA)

High between-study heterogeneity was observed under five models. Meta-regression suggested that ethnicity (*P* = 0.163), HWE (*P* = 0.621) and genotyping method (*P* = 0.514) had no significant impact on the heterogeneity in ORs with *IL-10* rs180086 polymorphism except for SOC (*P* = 0.043). Consequently, SOC may be the main source of high heterogeneity. We assessed the sensitivity by omitting each study in turn to assess the effect of individual study on the overall estimated risk. The pooled ORs did not show a significant difference by removing any individual study, supporting that this meta-analysis was stable and trustworthy (GA vs. AA, Figure 4). Both Egger’s and Begg’s tests (GG vs. GA+AA, Figure 5) were used to evaluate the publication bias of this meta-analysis. No evidence of publication bias was found in this meta-analysis (Table 2).

## DISCUSSION

The role of *IL-10* in tumor pathogenesis and development is a double-edged sword. Many studies have revealed the significant association between *IL-10* levels and poor prognosis. Moreover, *IL-10* is also thought to promote tumor immune escape by diminishing anti-tumor immune response in the tumor microenvironment [28]. Richter et al. found *IL-10* showed the anti-tumor effect on the suppression of angiogenesis and metastasis [9]. Notably, rs1800896 polymorphism in the *IL-10* gene promoter could affect *IL-10* production [29]. Recently, the association between the *IL-10* rs180086 polymorphism and various cancers has been reported widely, including esophageal cancer [30], oral cancer [31], gastric cancer [32] and colorectal cancer [33]. Of course, PCa is also among these cancers that could not be ignored. In 2002, McCarron et al. found that *IL-10* rs1800896 polymorphism could decrease the risk of PCa in the United Kingdom [19]. The significant association was confirmed in the subsequent studies among Caucasians [16, 22]. However, the remaining studies failed to uncover the association between the *IL-10* rs1800896 polymorphism and PCa risk in different races [5, 13-15, 17, 18, 20, 21, 23-25, 27].

To address this controversy, Shao et al. reviewed 10 studies involving 3,451 cases and 4,440 controls and performed a comprehensive meta-analysis in 2011 [34]. They found that there was no significant association between the *IL-10* rs1800896 polymorphism and the risk of PCa in the overall population [34]. In the subgroup analysis, negative results were also found among Caucasians and Asians [34]. In another meta-analysis involving 4846 cases and 5244 controls from 12 studies, Zou et al. [35] got the same findings as the meta-analysis conducted by Shao et al. [34]. In 2017, Ruan et al. [36] found that *IL-10* rs1800896 polymorphism was not associated with the risk of PCa in overall populations. However, they found rs1800896 polymorphism might increase the risk of PCa among Asians in the stratification analysis of ethnicity [36]. In this study, we re-evaluated the role of *IL-10* rs1800896 polymorphism in the risk of PCa by meta-analysis involving 12,811 subjects. Our data found that *IL-10* rs1800896 polymorphism was associated with the decreased risk of PCa. In addition, we think previous meta-analyses [34–36] had several limitations. First, Shao et al. [34] did not conduct a comprehensive research and omitted an article [5]. Second, Zou et al. [35] did not analyze the source of heterogeneity and conduct sensitive analysis to evaluate whether individual study could influence the overall result qualitatively. Third, Ruan et al. [36] did not include three studies [22, 25, 37] which actually conformed to the inclusion criteria. In addition, Ruan et al. extracted wrong genotype number of rs1800896 polymorphism from the study conducted by Wang et al. [16]. Consequently, the reliability of their results [34–36] should be interpreted with caution. We believe our meta-analysis has some strength over previous meta-analyses for the following reasons. One, we identified 16 studies including 6,301 cases and 6,510 controls with regard to rs1800896 polymorphism and the sample size of this meta-analysis was large enough. Two, sensitivity analysis indicated that our data about rs1800896 polymorphism were trustworthy and robust.

Recently, a study found *IL-10* can completely prevent antigen-specific T cell proliferation by inhibition of the antigen-presenting capacity of monocytes through downregulation of class II major histocompatibility complex (MHC) antigens on monocytes [38]. The finding suggested a mechanism for tumor cells escaping from immune surveillance. In addition, AA homozygotes of rs1800896 polymorphism in the *IL-10* gene could reduce the production of *IL-10* [39]. Based on these observations, we guessed that less *IL-10* was beneficial for immunological monitoring, reducing the possibility of tumor cell survival. It may partly explain the reason why *IL-10* gene rs1800896 polymorphism was associated with the decreased risk of PCa.

The stratified analyses further confirmed this significant association in population-based studies and studies using the genotyping method of MassARRAY. In addition, stratification analysis by ethnicity indicated that the G allele of rs1800896 could decrease the risk of PCa among Caucasians (11 studies involving 4,438 cases and 3,600 controls) and increase the risk of PCa among Asians (2 studies containing 360 cases and 358 controls), indicating different racial inheritance of Caucasians and Asians. This discrepancy may partly attribute to ethnicity-specific effect and sample sizes. For Caucasians, the minor allele G frequency was 0.467 in this meta-analysis, which was much higher than that of Asians (0.164). We hypothesized that genetic heterogeneity, clinical heterogeneity, different genotyping methods and random errors may also be the potential reasons for different findings between Asians and Caucasians. In addition, sample sizes in the subgroup of Asians were relatively small, thus the findings of Asians may be underpowered. Consequently, the results of Asian groups need be interpreted with caution. Further studies with larger sample sizes is necessary to confirm these hypotheses.

Multiple meta-analyses were conducted to investigate the association between *IL-10* rs1800896 polymorphism and cancer risk [40-42]. The meta-analysis conducted by Dai et al. found that *IL-10* rs1800896 polymorphism had no association with breast cancer risk in the overall population [41]. However, G allele of rs1800896 was demonstrated to significantly increase susceptibility to gastric cancer and digestive cancer [40, 42]. In this meta-analysis, we obtained an association between *IL-10* rs1800896 polymorphism and the decreased risk of PCa. In summary, the above findings may suggest that *IL-10* rs1800896 polymorphism has disease-dependent functionality, which may explain the inherent heterogeneity of tumor progression in different types of cancer.

Several potential limitations should be addressed in this meta-analysis. First, no further stratification analyses of other potential factors, such as age and familiar history due to lack of original data. Second, our results were based on unadjusted estimates for confounding factors, which might have affected the final conclusions. Third, the sample sizes of some individual studies were very small, which could deviate from the true results. Fourth, the results should be interpreted with caution due to high heterogeneity. Fifth, some bias may be unavoidable because only articles published in English and Chinese were identified in this study. Finally, the sample sizes of Asian groups were limited.

In conclusion, this meta-analysis indicates that *IL-10* gene rs1800896 polymorphism is associated with the decreased risk of PCa. *IL-10* gene may have an ethnicity-specific effect and disease-dependent functionality. Larger studies with more diverse ethnic populations are needed to confirm these results of this meta-analysis.

## MATERIALS AND METHODS

### Search strategy and selection criteria

PubMed, EMBASE, Google and CNKI databases were searched for all relevant articles up to April 1, 2017. The following search terms were used: (“prostate cancer” OR “prostatic neoplasm” OR “prostate carcinoma” OR “prostate neoplasms”) AND (“polymorphism” OR “polymorphisms” OR “variant” OR “SNP” OR “SNPs” OR “rs1800896”) AND (“Interleukin 10” OR “*IL-10*” OR “IL10” OR “Cytokine Synthesis Inhibitory Factor”). References in cited studies and review articles were also checked for other relevant articles. We included the latest study when one study overlapped with another one. PCa was diagnosed according to classification criteria.

Studies were included if they: (1) discussed the association between the *IL-10* rs1800896 variant and PCa; (2) provided sufficient data for calculating the pooled odds ratio (ORs) with 95% confidence interval (CI); (3) were case-control studies. Exclusion criteria were: (1) studies containing overlapping data; (2) case reports or reviews; (3) articles investigating other SNPs of *IL-10* gene.

### Data extraction and quality assessment

Two investigators extracted data from the original studies independently. From each study, the following information was extracted: name of the first author, publication year, country of origin, ethnicity, genotypes of cases and controls, and genotyping method. Authors were contacted to provide supplemental data if data were not available in the relevant articles.

The Newcastle-Ottawa Scales (NOS) were used to assess the quality of the selected studies [43]. Total NOS scores ranged from 0 (worst) to 9 (best). Only the studies with more than 5 stars based on the NOS scale were included in this meta-analysis. The discrepancies were resolved by consensus.

### Evaluations of statistical associations

Pearson’s χ2 test was used to evaluate whether gene frequencies in the controls conformed to HWE. Pooled ORs with corresponding 95% CIs were calculated to evaluate the strength of the relationship between *IL-10* rs1800896 polymorphism and the risk of PCa. Stratification analyses were carried out by ethnicity, SOC, HWE and genotyping method. Heterogeneity between studies was detected by Cochran’s Q-statistics. Generally, we considered the presence of significant heterogeneity at the 10% level of significance and values of I^2^ exceeding 50% as an indicator of significant heterogeneity. When no heterogeneity was found with *P* > 0.10 or I^2^ < 50%, a fixed-effect model was used. Otherwise, a random-effects model was applied [44]. To detect potential between-study heterogeneity, meta-regression was performed using the following covariates: ethnicity, SOC, HWE and genotyping methods. Sensitivity analysis was conducted to determine the effect of each study on the pooled ORs by omitting each study in turn. Publication bias was evaluated by visual inspection of symmetry of Begg’s funnel plot and assessment of Egger’s test [45]; *P* < 0.05 was regarded as representative of statistical significance. All statistical analyses were performed using the Stata 11.0 software (StataCorp, College Station, TX, USA).
